# Policymakers and mHealth: roles and expectations, with observations from Ethiopia, Ghana and Sweden

**DOI:** 10.1080/16549716.2017.1337356

**Published:** 2017-08-25

**Authors:** Catharina Barkman, Lars Weinehall

**Affiliations:** ^a^ Forum for Health Policy, Stockholm, Sweden; ^b^ Epidemiology and Global Health, Department for Public Health and Clinical Medicine, Umeå University, Umeå, Sweden

**Keywords:** mHealth for Improved Access and Equity in Health Care, eHealth, ICT, policymaking, upscaling, implementation

## Abstract

The rapid increase in mobile phone use and other telecommunication technologies in health care during the past decade has paved the way for optimism. mHealth (mobile health) initiatives need to be integrated into national health systems and priorities and fit into the system that the country has already invested in. Partnership between government, regional governments, health care systems, Community Health Workers, the private sector and universities is considered as a precondition for success. In turn, this requires strategic and integrative policy decisions on the national/regional level to be defined in the action plans as concrete steps. Decision makers are calling for scale-up plans to be in place even in the pilot phases. Hope is expressed that the initial joy and curiosity that new technology generates in the implementation phase will be transferred to routine work. Standards and a common technical architecture that enables interoperability and upscaling are key issues. Based on publications on policy and national strategies, this paper highlights some key areas for decision makers’ role and expectations with regard to mHealth. The paper will also report some mHealth experiences from Ethiopia, Ghana and Sweden.

## Background

mHealth is:

a service or application that involves voice or data communication for health purposes between a central point and remote locations. It includes telehealth (or eHealth) applications if delivery over a mobile network adds utility to the application. It also includes the use of mobile phones and other devices as platforms for local health-related purposes as long as there is some use of a network. [1]

In this paper, we do not differentiate between eHealth and mHealth since both are used in describing digital health care development.

Creative mHealth applications are able to transform health services in low-, medium- and high-income countries by, among other things, bringing health care to unserved or underserved populations [2]. A systematic digitalization of the health care system could lead to a more sustainable cost trajectory and could also improve the quality of care. New mHealth applications are emerging as ways to address contemporary health challenges in a better way [3,4].

Mobile phones can create entirely new opportunities for health care, especially in many low- and middle-income countries (LMIC) with shortcomings in infrastructure, expertise and human resources in the health care system. As new studies add further experiences on the value of mobile information and communication solutions globally, awareness is increasing of its potentials among practitioners, researchers and decision makers. In many LMIC, confidence is growing that mHealth solutions could alleviate the problems of health systems caused by under-funding, lack of qualified staff and inefficient procedures [5].

The rapid increase in mobile phone use and other telecommunication technologies during the past decade has paved the way for this optimism. It has been shown that digitalization can drive improvement in health care processes and organizations [6].

mHealth studies in LMIC illustrate a burgeoning development of knowledge in the field. However, the results of mHealth studies have not yet had a significant impact on the countries’ policies and investments in mHealth [7].

History shows that the development of eHealth/mHealth infrastructure is not without difficulties [8]. Many countries invest heavily in different kinds of IT systems that prove to be administratively complex and not always successfully deployed. The focus in these cases is mainly on the technical aspects and not on the patient’s or doctor’s needs. In contrast, private companies (e.g. Apple, Microsoft, Google, Spotify) build their initial digital products and services based on customer surveys and then develop their offerings. Another barrier in some countries is the lack of cooperation between the public health sector, private business and research [9].

The aim of this paper is to explore policymakers’ roles and expectations with regard to mHealth/eHealth with the main focus being on LMIC-relevant aspects. We illustrate some important topics by reporting mHealth policies in Ethiopia and Ghana. We also highlight some principal issues from the experiences in Sweden to illustrate the digital development in a developed country.

## Decision makers’ responsibility

The political responsibility is to create optimal conditions for mHealth implementation, in terms of both infrastructure and regulatory frameworks. This will in turn support confidentiality and also shape the finance and reimbursement models. It is essential that politicians establish a framework for university–public sector–industry relationships as well as clear rules for participation from the private sector to facilitate private partners entering the market [10].

A reasonable explanation for why mHealth is not yet given a higher priority on the political agenda might be that the evidence of its scalability and long-term impact on health outcomes and cost benefits is so far insufficient [8]. This could lead to insufficient levels of financial investments in mHealth and IT. The impact of mHealth projects should be judged on how the technology influences people’s behaviour (both patients and professional health care workers) to improve the health service [11].

Of particular importance is whether or not the mHealth solutions take into account *local needs*, as mHealth solutions must fit in resource-poor settings. Many times technology solutions designed for rural need also to fit urban areas. But it may be difficult to adapt urban solutions to fit rural needs [5,11].

A particular challenge for policymakers is to identify *financial/reimbursement models* that support development of mHealth from pilot tests to the system level [7].

Credence would be lent to mHealth legitimacy by transparently monitoring and evaluating contributions of improved work processes, developed service efficiency, improved patient safety and improved health outcomes [12].

## mHealth solutions

Governments’ and national administrations’ roles are to develop mHealth strategies and coordinate the objectives of mHealth with defined national policies. Politicians must allow professionals, researchers and private operators, who have the necessary creativity and foresight, to find new solutions. How these two preconditions can connect and work together is what fosters development in mHealth [10,13].

It is a great advantage with regard to both safety and cost if new solutions take advantage of, and build on, an *existing technical infrastructure* [3]. If these criteria are met the solutions will receive growing interest from decision makers [7].

The development and distribution of *generic service platforms* also play a vital role. These provide the processing power, storage, security, access control and other services to a broad range of mobile applications, including mHealth. When these services are available as platforms everybody can use, in the form of mobile networks or the Internet, the marginal cost required to develop new applications will be significantly lower [1].

Another important factor is *improved education and training* in the field of eHealth and mHealth for medical personnel. One bottleneck, even with today’s technical infrastructure, is the lack of knowledge among doctors and nurses of how to maximize the potential of existing systems, not to mention understanding and learning new mobile applications [5,14].

## Coordination and partnerships

Lower costs and better network coverage create greater opportunities for a wider range of applications based on mobile phones and other telecommunication technologies, which in turn increases the possibilities of using mHealth in health care [14].

In developing countries, mobile infrastructure has higher penetration than fixed networks. In many places, the only technical possibility for mHealth solutions is digital infrastructure such as flexible cloud solutions and mobiles. Data-carrying capacity of mobile networks is increasing rapidly and can often support programs needed in the medical field, to transmit high-resolution images, videos and large files. Simple mobile phones are, via text messaging, powerful tools. Smartphones, however, have much greater computing power, potential for data storage, and can also create opportunities for interaction with sensors and an intuitive user interface, which in turn can be used as a platform in many kinds of high-tech solutions [1].

mHealth applications are also used for training and decision support via automated analysis of data or real time in consultation with specialists. This makes it possible for local health workers to diagnose and treat conditions remotely without patients needing to travel to specialized hospitals far away from their homes.

It is an urgent task to develop generic mHealth models that support mHealth solutions and fit different health conditions or primary care needs. Even if a research program based on mHealth applications focuses on a particular disease, such as HIV/AIDS, it is necessary that the mHealth application also be useful for health interventions in general. Electronic medical records software or a specific mobile application must be able to communicate with other systems [13].

Investments in publicly available services have the potential to contribute to several programs. The more programs that are involved, the more profitable the investment. One advantage is that operators do not need to go into the health sector, as they only need to activate the application to be used. This can in turn facilitate sector-driven innovation, and makes it possible to develop applications even if the user base is small [1].

## Standardization

Reports from both ministries and international agencies state that standardization is a key issue. The World Health Organization (WHO) is by many seen as the ‘technical agency’, which can help to deliver the best buys/best practices for mHealth. There is a strong need for a common technical architecture that permits interoperability and upgrading, while at the same time, standards are also needed that clarify national ownership, control of business rules and information flow [13].

In consultation with market participants, the role of governments and national administrations must be to find a balance between the individual solutions versus standardization. Without standardization comes the risk of chaos and inefficiency. On the other hand, excessive standardization could paralyze development by removing business incentives.

It is imperative that mHealth solutions are sustainable. A partnership between the government, local authorities, health care systems, universities, private sector (e.g. digital platforms) and donors is essential in the planning phase in order to identify common goals. mHealth requires strategic, integrated national efforts based on (if possible) common goals. These must be adopted by the parties concerned and outline the main mission and policies, including concrete steps and follow-up plans. The private sector should be involved from the start, represented by both large and small companies and also start-ups [5,10].

Coordination between ministries/national authorities, academia, health care providers and private business is seen as a prerequisite for success. Forums, both digital and physical, promote dialogue within partnerships and new ideas.

Within the national authorities, it is particularly important for mHealth development to occur within a consensus between the Ministry of Health and Ministry of IT and Telecoms [5]. This must include common policies, effective utilization of common resources, partnerships with the private sector and upgrading mHealth competence within the health service [14]. Solutions must be designed in conjunction with Community Health Workers, who constitute the backbone of the health care system and guarantee acceptance among patients and the general public [14].

Expectations of potential benefits have caused more than 100 countries to explore mHealth as a means of achieving better health. At the same time, there is widespread recognition that there are also many obstacles and challenges to be addressed in order to achieve success, such as limitations of access, as well as health and technological illiteracy.

At an mHealth symposium, ‘Evidence from low- and middle-income countries’, organized in 2015 by UCL Institute for Global Health, BBC Media Action and Umeå Centre for Global Health Research, Sweden, a number of challenges for mHealth interventions were summarized. These included:

a lack of national policies to inform decision making, a lack of mHealth initiatives at a national scale, limited integrated partnerships between national governments and commercial organizations, limited attention on how to tackle ethical issues around consent, privacy and data protection and the separation of mHealth interventions from existing health systems. [15]

## Examples from three countries

Ethiopia, Ghana and Sweden have different experiences with the introduction of mHealth in their health systems. Without claiming completeness, we summarize the efforts and experiences of each country.

### Ethiopia

Ethiopia currently has over 90 million inhabitants, 80% of whom live in rural areas. The country has a decentralized three-level system of primary, secondary and tertiary care where the regional and district levels have great influence.

The lowest level in Ethiopia’s health system is the primary health care unit, which usually consists of five ‘health posts’, one health centre and one primary hospital. In total there are 17,000 health posts. Each health post has two Health Extension Workers (HEW) who provide preventive and basic curative services. These HEW represent the backbone of the health system. A HEW has one year of health training, and is employed and paid by the state. A HEW is also part of a career system which can provide opportunities for training and advancement. HEWs not only provide skills and knowledge of health, but must also be agents for social transformation. For example, they work with the Model Family Training Programs which train families on issues of importance to individuals’ health, and, according to the model of diffusion, allow these families to serve as models in the local community.

Health centres have around 20 health professionals and are responsible for the preventive, curative, inpatient and ambulatory services, treatment of common psychiatric disorders, and dental services. A primary hospital provides approximately 60,000–100,000 people with preventive, curative, inpatient, ambulatory and emergency surgical services, including caesarean section and blood transfusion. They serve as referral centres for the health centres and as training centres for nurses and paramedical health professionals. The next level above the primary hospital, called a General Hospital, serves about 1–1.5 million people, while each of the 28 Specialized Hospitals has a catchment area of 3–5 million people.

For Ethiopia, the development of mHealth is considered to be of particular importance within five areas:

- Data exchange to assess whether HEWs reach their community
- Supply chain so that the pharmaceutical supply is guaranteed
- Real-time referral to the next higher level of care
- Consultations between hospitals and remote area services
- Training and health education, for acquiring skills locally.

mHealth solutions are regarded by the Ministry of Health to be important resources at the health centre level, which is why staff at this level should have specialized expertise in information and communication technology (ICT). Therefore, priority is given to employment of Health Information Technicians (who undertake three years of education after high school) at every health centre in Ethiopia. These technicians have a mandate to (a) improve the computer skills of the staff in the unit, (b) report health data upwards in the system and (c) extract health data for local use to improve the quality of care. Both Primary and General Hospitals are also staffed with Health Information Technicians. The national goal is to have 10,000 technicians with this needed competence by 2020.

Parallel to this, a Public Health Emergency Management System has been developed to report daily or weekly on 23 severe diseases. In addition, a Drug Supply Management System has been implemented, with 26 regional hubs, that is responsible for keeping track of drug availability at all health centres.

Ethiopia has more than 33 million mobile phones, mainly basic models. However, progress towards smartphones has been rapid. Thus, mHealth solutions must take into account both the need for appropriate technology and also adherence to country standards, while at the same time promoting the desired health service outcomes [15,16].

### Ghana

Ghana currently has about 26 million inhabitants, of whom almost 50% live in rural areas. In 2007, Ghana adopted a new National Health Policy. Subsequently, however, it became increasingly evident that the health sector needed new and innovative ways of reaching more people with information and resources to help them make informed decisions. This led the Ministry of Health (MoH) to adopt a National e-Health Strategy in 2010, with four strategies:

(1) Streamlining the regulatory framework for health data and information management, (2) Building sector capacity for wider application of e-health solutions in the health sector, (3) Increasing access and bridging equity gap in the health sector through the use of Information and Communication Technology, and (4) Towards a paperless records and reporting system. [17]

mHealth represents a key future component of this national strategy, which has the following goals:

- Mobile phone service to provide engagement to meet overall health sector objectives.
- Appointment of an interagency team to assess how specific services to support treatment and follow-up, adherence to medication and patient support could be developed with mobile phones.
- Establishment of disease surveillance and epidemic tracking systems within the Ghana Health Service which use mobile telephones and involve the private sector.
- Provision of real-time information for selected diseases [17].

This national strategy proposes action areas as pilot stages as well as a mechanism for scaling up where necessary. The strategy expects the government and private actors to work together to develop and implement new solutions and to share the added value of what they jointly achieve. The government will define standards and rules on data protection, and introduce mechanisms for implementation and enforcement. Special attention will be given to innovative solutions that in the near future can contribute to improved public health [17].

Specific eHealth solutions will predominantly be driven by the MoH in collaboration with stakeholders and solution vendors which will enable the development of eHealth solutions that meet specific health sector needs. Through the MoH, the government has the overall responsibility for setting the national eHealth agenda and is directly responsible for funding, implementing and operating eHealth infrastructure. It is hoped that this will stimulate and encourage the market to develop quality eHealth solutions that are scalable, standards compliant and aligned with national priorities. To help keep the implementation on track, the strategy identified several critical building blocks for the first four years, including eHealth coordinating, regulatory frameworks, mHealth pilots, broadband connectivity, an eHealth human resource capacity development programme, a functional Telemedicine pilot, initiation of electronic public interaction with the health sector, an electronic patient records system piloted in selected health facilities and a Web-based District Health Information System [17].

Since 2010, several pilot mHealth projects, including Internet-based consultancy, have been initiated in Ghana. In a published review which illustrates the mHealth development in Ghana from 2010 to 2013, 22 pilot projects were identified at various stages of implementation [18]. However, in the most recently presented Health Sector Medio-Term Development Plan 2014–2017 from the Ghana MoH, the eHealth Strategy was not specifically mentioned in the text. This could possibly indicate that the landmarks identified as critical building blocks for the success of the eHealth strategy for the first four years are not yet fully in place [19]. Another possible explanation could be that the field studies currently are financed by donations, which is why these efforts have not yet become visible in the MoH’s budget.

### Sweden

Health care in Sweden is largely tax-funded, with responsibility for health and medical care shared by the central government, county councils and municipalities. The role of the central government is to establish principles and guidelines and implement laws and ordinances. The counties are responsible for providing health care and the municipalities for providing elderly care. Challenges include future funding (demographic development), quality (large variations in the country) and efficiency (results clearly indicate potential for improvement).

The central government and the Swedish Association of Local Authorities and Regions have endorsed a shared vision [20]. Many activities related to digitization of mHealth/eHealth at various levels are already under way. More than 90% of pharmaceutical prescriptions are e-prescriptions (electronically transmitted prescriptions) which are generated in the doctors’ electronic prescription system and transmitted through a secure network to the national e-prescription database. Patients can pick up their medication when they choose at any pharmacy throughout the country. Through the platform My Health Contacts (Mina VårdKontakter: https://www.1177.se/Vasterbotten/Other-languages/Engelska/), a patient can use their mobile phones to request, reschedule or cancel appointments, request prescriptions and ask to be contacted by a health care centre.

Even though Sweden has long experience in digital development in general, the development in mHealth/eHealth is relatively slow. A number of bottlenecks constrain how fast new technologies are implemented and what benefits they yield. Thus, there are some lessons to be learned on what key elements are necessary for success:

- Adoption of necessary legal changes to make digital documents equally valid with paper documents and to ensure security and privacy rules etc. (In Sweden the patient has the right to see their own medical records, decide on information sharing and block access to information.) Transparency of health data for the patients is also essential from a democratic aspect.
- Agreement among key parties in the health care sector about common use of terminologies and codes to ensure standardized data for research and development.
- Focus on changing processes in health care when implementing new technology. When medical records were first computerized in Sweden, the administrative burden for doctors increased. The main reason for this was that routines for writing medical journals did not change. Demands increased on doctors and nurses to report more data. Today there is too much documentation in health care which jeopardizes patient security. Swedish doctors spend 60% of their working hours with patients [21].
- Infrastructure for mHealth/eHealth. Use what is already working and available. One example of this is mobile ID in Sweden which was introduced by banks and is now applicable in health care.
- Develop reimbursement systems/financing systems that enhance mHealth/eHealth development. For example, in some areas in Sweden, doctors only get paid if they see the patient in person, not online.
- Set up clear governance to ensure possibilities for private entrepreneurs. Governance should focus on infrastructure and standardization, and free up private business to develop IT applications through an authorization process.
- Prioritize financial investments in mHealth/eHealth. In Sweden the IT share of the health care budget has been constant at a level of 2.83% since 2003, while eHealth/mHealth development and IT users have increased by 90% [22].
- Focus on evaluation when installing or testing new mHealth applications.

Digitalization of health care in Sweden faces many challenges, including integration of health data collected by individuals using either remote monitoring systems or mobile devices, use of digital decision support to develop personalized medicine, and implementation of new e-services (specially to integrate social care and health care services).

## Summary

Digitalization in health care drives patient-centred improvement and may increase efficiency and improve quality of care. As previously presented, Ethiopia has focused on mHealth development, predominantly in rural areas. The country’s plan is to staff every health centre, primary hospital and General Hospital with trained Health Information Technicians. As Ethiopia already has 33 million mobile phone holders, there is great potential to strengthen mHealth development. Ghana adopted a National e-Health Strategy in 2010 with a priority to serve rural areas. Since then a large number of pilot mHealth projects, including Internet-based consultancy, have been initiated in the country. Sweden has a high level of digitalization, but the road has been rocky. Many lessons have been learned as to the importance of introducing reimbursement models that support innovation and mHealth development, setting up clear governance to facilitate cooperation with both research and private business, and finding a balance between standardization of terminology and codes on the one hand and innovation and development on the other.

Successful development of mHealth requires clear-cut roles for key parties. Governments’ and national administrations’ main role should be to create prerequisites in a proper way for professionals, researchers and private operators with creativity and foresight to find new solutions.

## Conclusions

To achieve good results, mHealth applications must interact with established health systems (and be regulated to fit these systems), which in turn is significantly influenced by how well the health care system can adapt to, and interact with, new technology. Research is essential to provide evidence-based findings on the results of governmental decisions. Private business plays an important role in creating new techniques, treatments and pharmaceuticals. The government’s responsibility is to create optimal conditions for mHealth to succeed, in terms of infrastructure, regulatory frameworks and reimbursement models. The government’s role is also to evaluate and transform successful pilot programs into full-scale implementation. The challenge is to balance these factors optimally.

In recent years, ministries, national authorities and international agencies of various countries have presented their assessments and expectations for mHealth implementation. There are numerous challenges, and it is always easier to formulate a policy than to implement it. However, by learning from other countries’ mistakes and successes, it is possible to speed up mHealth development.

## Paper context

To be sustainable, mHealth (mobile health) initiatives need to be integrated into national health systems and priorities and fit into the system that the country has already invested in. Strategic and integrative policy decisions on the national/regional level are required in the concrete steps of action plans. Partnership between government, health care providers, Community Health Workers, the private sector and universities is considered a precondition for success.
